# Late presentation of hepatitis B among patients with newly diagnosed hepatocellular carcinoma: a national cohort study

**DOI:** 10.1186/s12885-019-5508-5

**Published:** 2019-03-29

**Authors:** Dong Hyun Sinn, Danbee Kang, Minwoong Kang, Seung Woon Paik, Eliseo Guallar, Juhee Cho, Geum-Youn Gwak

**Affiliations:** 1 Department of Medicine, Samsung Medical Center, Sungkyunkwan University School of Medicine, 81 Irwon-ro, Gangnam-gu, Seoul, 06351 South Korea; 2 Department of Clinical Research Design and Evaluation, SAIHST, Sungkyunkwan University, 81 Irwon-ro, Gangnam-gu, Seoul, 06351 South Korea; 3 Center for Clinical Epidemiology, Samsung Medical Center, Sungkyunkwan University, Seoul, South Korea; 4 Departments of Epidemiology and Medicine and Welch Center for Prevention, Epidemiology and Clinical Research, Johns Hopkins Medical Institution, Baltimore, MD USA

**Keywords:** Hepatitis B, Hepatocellular carcinoma, Late presentation, Mortality

## Abstract

**Background:**

Recently, the concept of “late presentation with viral hepatitis” was introduced to help quantify the proportion of patients missing timely diagnosis and treatment for viral hepatitis. The clinical implications of late presentation of hepatitis B at the population level, however, are largely unexplored.

**Methods:**

Using newly-diagnosed hepatitis B related hepatocellular carcinoma (HCC) patients (*N* = 1276) from the Korean National Health Insurance Service-National Sample Cohort, a nationally representative cohort study was conducted between 2002 and 2013. HCC patients were classified into 3 groups: late presentation of hepatitis B (no prior clinic visits for hepatitis B before HCC diagnosis), irregular visits (irregular pattern of outpatient clinic visits), and regular visits (regular pattern of outpatient clinic visits).

**Results:**

The proportion of patients with late presentation decreased from 50.8% in 2003 to 23.1% in 2013. In multivariable analysis compared with patients in the regular visits group, patients with late presentation were more likely to be younger and to be in lower income percentiles. After adjusting for age, sex, year of HCC diagnosis, income percentile, and initial treatment, the hazard ratios (95% confidence intervals) for all-cause mortality comparing the late presentation and irregular visits groups to the regular visits group were 1.76 (1.42–2.18) and 1.31 (1.06–1.61), respectively.

**Conclusion:**

Timely diagnosis and treatment for hepatitis B related HCC was suboptimal at the population level. More intensive strategies to minimize late presentation for hepatitis B are needed, with special attention to younger people and lower income levels.

## Lay Summary

- Early identification of patients with hepatitis B can reduce burden from hepatitis B by providing effective antiviral treatment and HCC screening.
- Late presentation of hepatitis B, defined as HCC cases presenting without a prior clinic visit for hepatitis B, was still common in Korea by 2013 and it was associated with increased mortality.
- Younger age and lower income levels were associated with a higher risk of late presentation.
- Policy strategies to reduce late presentation of hepatitis B should be established to achieve optimal control of HCC.

## Background

Hepatocellular carcinoma (HCC) is one of the leading causes of cancer death globally [1] and a substantial contributor to the burden of disease worldwide. In contrast to other major types of cancer, approximately 80% of HCC cases have well-known etiologies such as hepatitis B virus (HBV) infection, hepatitis C virus (HCV) infection, or alcohol use [2, 3]. Furthermore, effective and well-tolerated treatments for HBV and HCV infections are available, which may decrease the risk of developing HCC [4–6]. Thus, early identification of patients with viral hepatitis could effectively contribute to the global control of HCC.

HBV infection is usually asymptomatic until the disease progresses to a late stage [7]. Consequently, a large but unknown proportion of subjects with chronic HBV infection learn of their infection and enter comprehensive care only after developing liver disease-related symptoms [8–10]. Routine testing to identify patients with chronic HBV infection at an early stage is recommended for high-risk populations, such as persons born in regions of high and intermediate HBV endemicity, pregnant women, and household, needle-sharing, or sexual partners of persons who have tested positive for hepatitis B surface antigen (HBsAg) [11]. However, screening recommendations are often not followed [12, 13], and it is difficult to estimate how well they are implemented in clinical practice. Recently, a European consensus working group introduced the concept of “late presentation with viral hepatitis” to help quantify the proportion of patients missing timely diagnosis and treatment. Late presentation with viral hepatitis was defined as patients who were not known to be infected previously but presented with symptoms of cirrhosis and/or HCC for medical care [10]. The clinical implications of late presentation of hepatitis B at the population level are largely unexplored.

In 1990s, the prevalence of HBsAg positivity was 8–10% in Korea. Because of high endemicity of HBV infection, several population wide-programs were implemented to reduce the burden of HBV infection and its consequences since the 1990s [3, 6]. These programs included universal HBV vaccination for newborns (started in 1995) [5, 6], a vertical transmission prevention program (started in 2002), free liver cancer screenings for individuals aged 40 years and older with HBV, HCV and cirrhosis (started in 2003) [6, 14], and a mandatory surveillance system for acute hepatitis B cases (started in 2010) [6]. Due to these programs, the prevalence of HBsAg positivity had been decreased to 2.9% in 2013 [6]. However, Korea is still classified as an area of intermediate endemicity for HBV [5, 6]. Furthermore, HBV and HCC screening practices in Korea are considered suboptimal [15] with many people unaware of their infection status [16]. We used a nationally representative sample to evaluate the proportion of patients with late presentation of hepatitis B among newly diagnosed HCC patients from 2003 to 2013 and its relationship with survival in Korea.

## Methods

### Study population and design

The National Health Insurance Service-National Sample Cohort (NHIS-NSC) is a population-based retrospective cohort comprised of a 2.2% representative sample of Korean citizens [17]. Korea has a single-payer national health system. The NHIS maintains national records of all covered inpatient and outpatient visits, procedures, and prescriptions as well as mortality data, and NHIS-NSC follow-up information is considered virtually complete. We used person-level longitudinal NHIS-NSC registration and claims data collected between January 1, 2002 and December 31, 2013 [17]. From this cohort, we identified 1393 patients with HBV infection who were 40 years of age or older, who had a new diagnosis of HCC between April 1, 2003 and December 31, 2013, and who had at least one year of medical records before HCC diagnosis. After we excluded participants with HCV co-infection (*n* = 117), the final sample size was 1276 (1020 men and 256 women). The Institutional Review Board of the Samsung Medical Center approved this study and waived the requirement for informed consent as we used only de-identified data.

## Data sources

The NHIS-NSC cohort comprises four databases on insurance eligibility, medical treatments, and medical care institutions. The insurance eligibility database contains information on age, sex, residential area, type of health insurance, income level, and disability. The medical treatment database contains information from treatment bills, including details of diseases and prescriptions. The general health examination database includes results from the health screening exams conducted by the NHIS [17].

NHIS claims for inpatient and outpatient visits, procedures and prescriptions were coded using the International Classification of Diseases, Tenth Revision (ICD-10) and the Korean Drug and Anatomical Therapeutic Chemical Codes [18]. The NHIS routinely audits the claims, and the data are considered reliable and have been used in numerous peer-reviewed publications [17, 19].

## Study variables

HCC was defined as three or more outpatient clinic visits with an associated C22.0 or C22.9 code within a year or one inpatient hospitalization with the same C codes. In Korea, once a person receives a cancer diagnosis, he/she is registered to the National Cancer Registry with a specific code (called C-code) that indicates to the system that the person has been diagnosed. C codes are carefully reviewed as they allow for additional insurance benefits for patients. C-codes are carried forward in medical records and claims created for the patient. Therefore, HCC and other cancer diagnoses based on claims are considered reliable, and a one-year look back window to exclude patients with a prior diagnosis of cancer (C code) excludes patients with a prior diagnosis at any time, and not just patients who had a diagnosis of cancer in the prior year.

For each case, we defined the index date as 3 months before the date of start of services for the first claim including the HCC diagnosis. We defined late presentation of hepatitis B as patients without clinic visit for hepatitis B (ICD-10 codes B180, B181, B1810, B1818, and Z225) prior to the index date [10]. Patients without late presentation of hepatitis B were further classified into: 1) irregular visits group: patients who had 1 or less clinic visit for hepatitis B in the 1 year period prior to the index date, and 2) regular visits group: patients who had 2 or more clinic visits for hepatitis B in the 1 year period prior to the index date.

Information on the underlying disease and comorbidities, demographics, income level, residential area, and initial treatment was based on claims codes. Income level was categorized as ≤30th, > 30th – ≤ 70th, and > 70th percentile. Residential area was classified as metropolitan or rural. Metropolitan areas were defined as Seoul, 6 metropolitan cities, and 15 cities that have a population > 500,000 and have been officially designated as municipal cities (http://www.mois.go.kr). Initial treatments for HCC were classified as liver transplantation, resection, radiofrequency ablation (RFA), transarterial chemoembolization (TACE), other, or no treatment.

## Statistical analysis

The outcome of the study was all-cause mortality. Person-time was calculated from the date of HCC diagnosis to the date of death or the end of the study period (December 31, 2013). Survival curves were generated by the Kaplan-Meier product-limit method and compared by log-rank tests. We calculated hazard ratios (HR) with 95% confidence intervals (CI) for all-cause mortality using Cox proportional hazards regression models. We used three models with increasing degrees of adjustment to account for potential confounding factors at the time of HCC diagnosis. These models were determined a priori based on biological and subject matter considerations. Model 1 was adjusted for sex, age group, and year of HCC diagnosis. Model 2 was further adjusted for income level category and residential area. Finally, model 3 was further adjusted for initial treatment. Since patient survival could be clustered by hospital, we used hospital as a stratification factor in Cox models. We examined the proportional hazards assumption using plots of the log (−log) survival function and Schoenfeld residuals.

In addition, we used multinomial logistic regression to identify factors associated with late presentation and with irregular visits using patients with regular visits as the base group. We considered a *P*-value of < .05 as statistically significant. All analyses were performed using STATA version 14 (StataCorp LP, College Station, TX, USA).

**Fig. 1:**
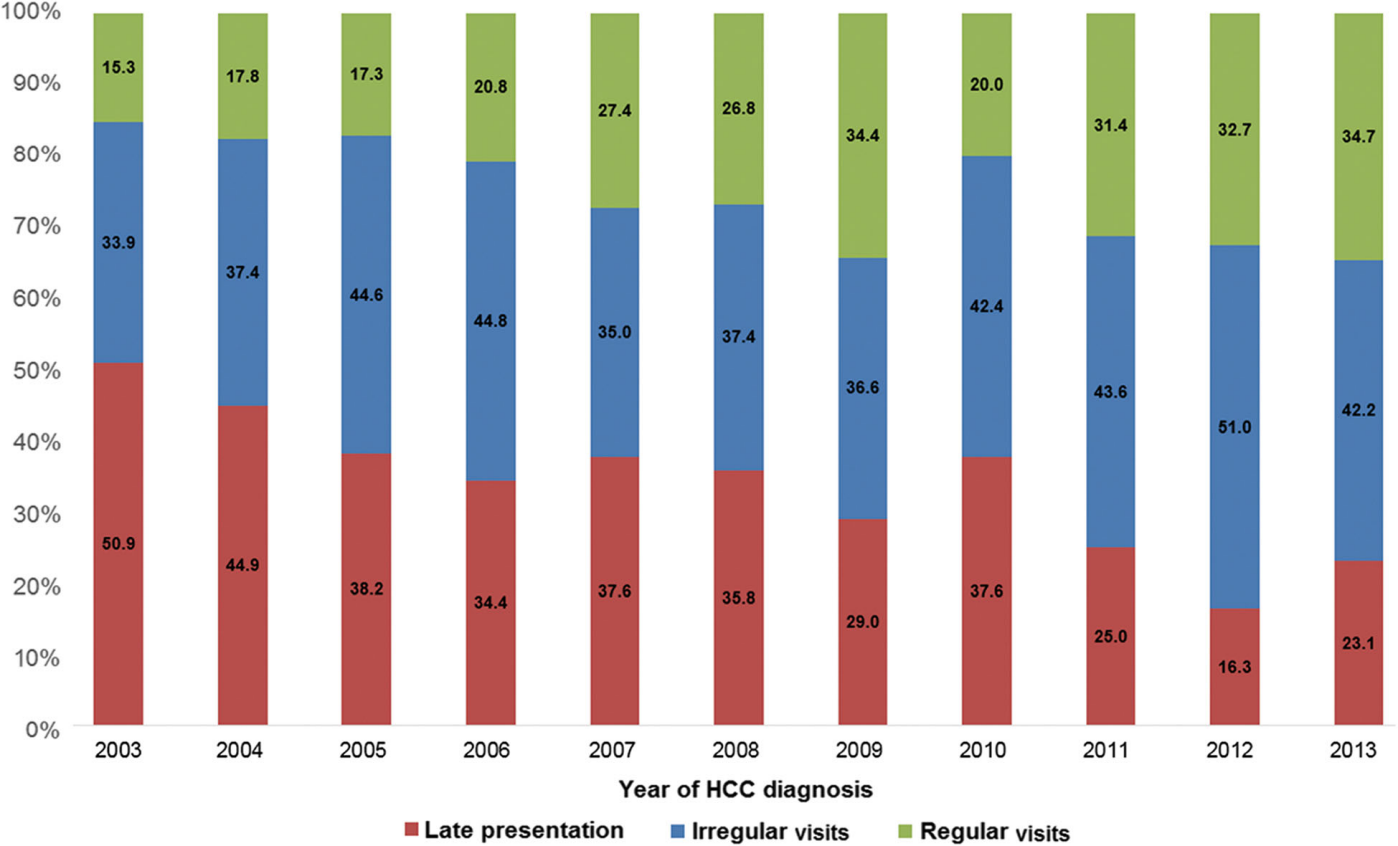
Proportions of patients with newly diagnosed hepatocellular carcinoma in the late presentation, irregular visits, and regular visits groups by year of diagnosis

Among 1276 patients who developed HCC related to HBV infection during the study period, 413 (32.4%) patients were in the late presentation group, 527 (41.3%) in the irregular visits group, and 336 (26.3%) in the regular visits group. Between 2003 and 2013, the proportion of patients with late presentation decreased from 50.8 to 23.1%, and the proportion of patients in the regular visits group increased from 15.3 to 34.7% (Fig. 1). The distributions of sex, age, and residential area were similar across presentation groups (Table 1), but the late presentation and the irregular visits group were more likely to have participants in the lowest income category compared to the regular visits group. Compared to the late presentation group, the irregular visits and the regular visits groups were also more likely to receive curative therapies for HCC, such as resection and RFA, as initial treatment.

**Table 1:** Characteristics of study patients at the time of diagnosis of hepatocellular carcinoma by hepatitis B presentation group

	Type of presentation			*p*-value
	Late presentation N (%)	Irregular visits N (%)	Regular visits N (%)	
Number of patients	413	527	336	
Sex				0.15
Male	343 (83.1)	415 (78.7)	262 (78.0)	
Female	70 (17.0)	112 (21.3)	74 (22.0)	
Age (years)				0.15
≥ 40 – < 50	105 (25.4)	106 (20.1)	64 (19.0)	
≥ 50 – < 60	165 (40.9)	230 (43.6)	163 (48.5)	
≥ 60	143 (34.6)	191 (36.2)	109 (32.4)	
Income percentile				< 0.001
≤ 30th	107 (25.9)	155 (29.4)	62 (18.5)	
> 30th – ≤ 70th	155 (37.5)	196 (37.2)	113 (33.6)	
> 70th	151 (36.6)	176 (33.4)	161 (47.9)	
Initial treatment				< 0.001
Liver transplantation	0 (0)	1 (0.2)	1 (0.3)	
Resection	40 (6.7)	59 (11.2)	60 (17.9)	
RFA	3 (0.7)	23 (4.4)	28 (8.3)	
TACE	193 (46.7)	219 (41.6)	140 (41.7)	
Other	95 (23.1)	116 (22.0)	50 (14.9)	
No treatment	82 (19.9)	109 (20.7)	57 (17.0)	
Residential area				0.12
Metropolitan	248 (60.1)	328 (62.2)	226 (67.3)	
Rural	165 (39.9)	199 (37.8)	110 (32.7)	

*RFA* radiofrequency ablation, *TACE* transarterial chemoembolization.Income percentile is based on household income

In multivariable analysis compared with patients in the regular visits group, patients with late presentation were more likely to be younger, and to be in the lower income category, and patients in the irregular visits group were more likely to be in lower income percentiles (Table 2).

**Table 2:** Multivariable-adjusted prevalence ratios (95% Confidence Interval) for factors associated with late presentation and irregular visits of hepatitis B in patients with newly diagnosed hepatocellular carcinoma

	Late presentation PR (95% CI)	Irregular visits PR (95% CI)
Sex		
Male	1.45 (0.99, 2.12)	1.09 (0.77, 1.68)
Female	Reference	Reference
Age (years)		
≥ 40 – < 50	1.53 (1.04, 2.26)	1.15 (0.79, 1.68)
≥ 50 – < 60	Reference	Reference
≥ 60	1.36 (0.96, 1.91)	1.26 (0.92, 1.73)
Income percentile		
≤ 30th	2.01 (1.36, 2.99)	2.35 (1.63, 3.40)
> 30th – ≤ 70th	1.56 (1.11, 2.18)	1.64 (1.19, 2.25)
> 70th	Reference	Reference
Residential area		
Metropolitan	Reference	Reference
Rural	1.28 (0.94, 1.74)	1.18 (0.88, 1.58)

Prevalence ratios (PR) were estimated using regular visits as the base group in multinomial logistic regression models adjusted for sex, age (≥ 40 – < 50, ≥ 50 – < 60, and ≥ 60 years), year of HCC diagnosis, income percentile (≤ 30th, > 30th – ≤ 70th, and > 70th), and residential area (metropolitan and rural)

**Fig. 2:**
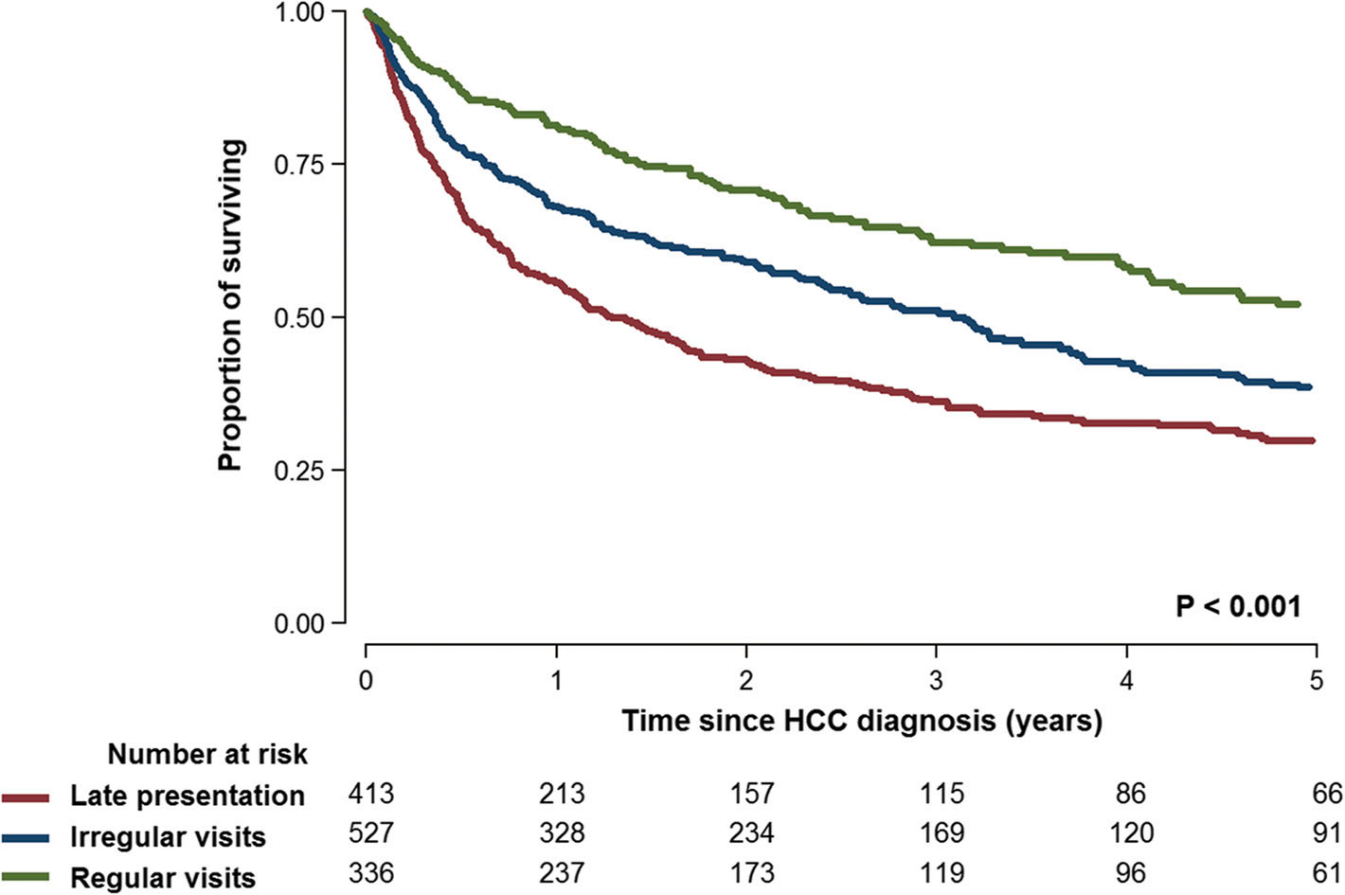
Kaplan-Meier survival curves for patients with newly diagnosed hepatocellular carcinoma in the late presentation, irregular visits, and regular visits groups

During 3234.1 person-years of follow-up (median follow-up 1.6 years; interquartile range 0.5–3.7 years), 685 patients died (274, 283, and 128 in the late presentation, irregular visits, and regular visits groups, respectively). Mortality rates were higher in the late presentation (28.7 deaths per 100 person-years) and the irregular visits groups (21.0 per 100 person-years) than in the regular visits group (13.7 per 100 person-year; Fig. 2 and Table 3). After adjusting for age, sex, year of HCC diagnosis, income percentile, and initial treatment, the hazard ratios (95% confidence intervals) for all-cause mortality comparing the late presentation and irregular visits groups to the regular visits group were 1.76 (1.42–2.18) and 1.31 (1.06–1.61), respectively (Table 3).

**Table 3:** Hazard ratios (95% confidence intervals) for all-cause mortality in patients with newly diagnosed hepatocellular carcinoma by hepatitis B presentation group

	Person-years	No. of cases	Incidence rate (per 100 person-years)	Crude HR (95% CI)	Model 1 HR (95% CI)	Model 2 HR (95% CI)	Model 3 HR (95% CI)
Late presentation	954.1	274	28.7	2.08 (1.69, 2.56)	1.99 (1.61, 2.46)	1.94 (1.57. 2.41)	1.76 (1.42. 2.18)
Irregular visits	1346.7	283	21.0	1.52 (1.24, 1.86)	1.49 (1.21, 1.82)	1.44 (1.17, 1.76)	1.31 (1.06, 1.61)
Regular visits	933.3	128	13.7	Reference	Reference	Reference	Reference
*p* value				<.001	<.001	<.001	<.001

*HR* hazard ratio, *CI* confidence interval.

*HRs were obtained from Cox models with hospital as a stratification factor.

Model 1: Adjusted for sex, age ((≥ 40 – < 50, ≥ 50 – < 60, and ≥ 60 years), and year of HCC diagnosis.

Model 2: Further adjusted for income percentile (≤ 30th, > 30th – ≤ 70th, and > 70th), and residential area (metropolitan and rural).

Model 3: Further adjusted for initial treatment (liver transplantation, resection, radiofrequency ablation, transarterial chemoembolization, other and no treatment)

## Discussion

In this nationally representative population-based study, the proportion of HCC patients with late presentation of hepatitis B declined from 2003 to 2013, although even in 2013 over 20% of newly diagnosed HCC cancer patients were in the late presentation group. Hepatitis B patients who were younger or in the bottom 30% of income had a higher risk of being late presenters. Patients with late presentation had almost twice the risk of mortality and those in the irregular visits group had 30% higher mortality compared to the regular visits group.

In our study, the proportion of patients with late presentation decreased over time, but only 35% of newly diagnosed HCC patients in 2013 were in the regular visits group and 42% were still in the irregular visits group. There still appears to be much room for improvement in screening and treating HBV in Korea. Additional strategies to improve screening and treatment of HBV in Korea include insurance coverage for antiviral prophylaxis in mothers with high viral load, screening for hepatitis B serological markers and catch-up HBV vaccinations in susceptible persons, management of HBV infection in immigrant populations, and national campaigns to promote awareness of hepatitis B [6].

Younger participants had a higher risk of late presentation in our study. This is consistent with a survey in Australia in which health behaviors significantly varied by age [20]. Compared to older people, younger people were less likely to seek health information and to undergo health screenings. Given that HBV-related HCC develops at younger age compared to other causes of HCC [21], and young HCC patients are more likely to be diagnosed at advanced stage compared to older patients [22], it would be important to increase awareness of the consequences of HBV infection among young people in Korea to promote testing for chronic infection and timely treatment and HCC screening.

Lower income was associated with a higher risk of late presentation in our study. Lower income was also associated with a lower likelihood of receiving regular HCC surveillance in cirrhosis patients in a study conducted in the United States [23]. Low income subjects may not be aware of the consequences of HBV infection and may not pursue routine screening testing. Furthermore, those tested positive may experience access barriers to regularly attend clinic visits for HBV treatment. We observed an income differential in the risk of late presentation in spite of a national health insurance system. Korea has a single-payer national health insurance system and it covers most costs associated with medical care, including treatments, for all citizens. Accordingly, costs for hepatitis B management are covered by the national health insurance and individual payment responsibility is the same for all citizens. Our findings may not generalize to countries with different health coverage or different individual payment responsibility for hepatitis B management.

Participants with late presentation or with irregular visits had worse survival than those with regular visits. Several reasons may explain this survival difference. Patients attending regular visits may be more likely to receive regular monitoring and treatment for HBV, which could decrease HCC risk by preserving liver function and improving outcomes [24–26]. Patients attending regular visits may also be more likely to undergo regular HCC screening. A recent study demonstrated that shorter HCC screening intervals are associated in a dose-dependent manner with reduced overall mortality in patients with HCC [27]. Indeed, high-risk patients receiving regular HCC screening are usually diagnosed at an early stage [28] and are more likely to be eligible for curative treatment. Consistent with this, patients in the regular visits group in our study were more likely to receive curative therapies including resection, transplantation and RFA compared to patients in the irregular visits or late presentation groups. Finally, patients in the regular visits group may be more likely to receive additional education for avoiding other liver toxins such as alcohol, further reducing their risk of HCC.

Several limitations need to be considered in the interpretation of our results. First, the differences in survival among patients in the regular visits, irregular visits, and late presentation groups may not be due only to beneficial effects of HCC screening and early intervention. In our study design, we cannot exclude the possibility of lead-time bias, as more frequent visits may just identify disease earlier, but not increase survival. In a study of 1380 patients with cirrhosis [29], lead-time bias explained some, but not all, survival benefit of HCC surveillance, which remained clinically useful even after taking lead-time bias into account. Although we cannot estimate the contribution of lead-time bias to survival differences, the survival disadvantage in late presentation and irregular visits patients provides important prognostic information as it identifies a large subgroup of patients with increased mortality over follow-up. Second, our data was based on administrative claims designed for reimbursement purposes, and did not include information on important factors that potentially affect survival of HCC patients, such as antiviral therapy for HBV, fibrosis stage, tumor stage, smoking, and drinking status. We also were not able to include some information that might be associated with late presentation such as knowledge or education level. Moreover, we did not have information on specific reason for late presentation (e.g., unawareness of HBV infection, lack of knowledge about the need for HCC surveillance, or misguidance by primary care physician, etc.). An additional limitation of our data was that we restricted our analysis to HCC patients while the definition of late presentation applies also to symptomatic cirrhosis patients [10]. This was because cirrhotic symptoms (e.g., ascites, variceal bleeding and hepatic encephalopathy) cannot be reliably identified using claims codes. Further studies are necessary to extend our findings to patients with cirrhosis symptoms. Finally, our study was conducted in Korea, a country with a single-payer health system, and our findings may not be generalizable to other countries with a different healthcare structure.

Regardless of these limitations, this is the first study to evaluate the impact of late presentation of hepatitis B on HCC mortality using national data over several years of follow-up. The relatively large sample size and the availability of data over an extended period of time also contribute to the strength of our data.

Several well-established prevention strategies, including HBV vaccination, HBV treatment, and HCC surveillance, decrease HBV-related HCC mortality [7]. However, the effectiveness of HCC prevention has been suboptimal at the population level [4]. Whereas the efficacy of interventions is primarily determined by its biological effects, its effectiveness can be influenced by various patient, provider, system, and societal factors such as identification of high-risk groups, availability and accessibility of care, acceptance and adherence to the interventions, and high-quality delivery of clinical services [4]. Our analysis justifies the need for more intensive HBV and HCC screening and treatment strategies to achieve optimal HCC control in a country of intermediate endemicity for HBV.

## Conclusions

Timely diagnosis and treatment for hepatitis B related HCC is still suboptimal at the population level in Korea. More intensive screening strategies to minimize late presentation for hepatitis B are needed, with special attention to younger people and lower income levels.

## Abbreviations

CI: Confidence interval
HBsAg: Hepatitis B surface antigen
HBV: Hepatitis B virus
HCC: Hepatocellular carcinoma
HCV: Hepatitis C virus
HR: Hazard ratio
ICD-10: International Classification of Diseases, Tenth Revision
NHIS-NSC: National Health Insurance Service-National Sample Cohort
PR: Prevalence ratio
RFA: Radiofrequency ablation
TACE: Transarterial chemoembolization
